# Computed tomography image quality of aortic stents in patients with aortic coarctation: a multicentre evaluation

**DOI:** 10.1186/s41747-018-0046-5

**Published:** 2018-07-18

**Authors:** Sara Boccalini, Annemarie M. den Harder, Maarten Witsenburg, Johannes P. J. M. Breur, Gabriel P. Krestin, Ingrid M. van Beynum, Mohamed Attrach, Nicola Stagnaro, Maurizio Marasini, Pim A. de Jong, Tim Leiner, Ricardo P. J. Budde

**Affiliations:** 1 Department of Radiology, Erasmus Medical Center, PO Box 2040, 3000CA Rotterdam, The Netherlands; 2 Department of Radiology, University Medical Center Utrecht, PO Box 85500, 3508GA Utrecht, The Netherlands; 3 Department of Cardiology, Erasmus Medical Center, PO Box 2040, 3000CA Rotterdam, The Netherlands; 4 Department of Pediatric Cardiology, University Medical Center Utrecht, PO Box 85500, 3508GA Utrecht, The Netherlands; 5 Department of Pediatric Cardiology, Erasmus Medical Center, PO Box 2040, 3000CA Rotterdam, The Netherlands; 6 Department of Radiology, IRCCS Istituto Giannina Gaslini, Via Gerolamo Gaslini 5, 16147 Genoa, Italy; 7 Department of Cardiology, IRCCS Istituto Giannina Gaslini, Via Gerolamo Gaslini 5, 16147 Genoa, Italy

**Keywords:** Aortic coarctation, Artifacts, Tomography, x-ray computed, Image quality, Stents

## Abstract

**Background:**

Stents are commonly used to treat aortic coarctation. The objective of this study was to evaluate the post-implantation computed tomography (CT) image quality of different stent types used to treat aortic coarctation.

**Methods:**

Adult and paediatric patients with stent-treated aortic coarctation who underwent contrast-enhanced CT were retrospectively included from three tertiary care centres. CT scans were subjectively scored for image quality using a 4-point scale (1 = unacceptable; 2 = poor; 3 = good; 4 = excellent). Furthermore, the amount of stent-induced blooming artefacts was measured as the percentage of the difference between outer and inner stent diameters over the outer stent diameter.

**Results:**

A total of 35 children and 34 adults implanted with 71 stents of six different types were included. The most commonly used stent type was the Cheatham Platinum stent (52 stents, 73%). The subjective image quality of the Cheatham Platinum stents was moderate with a score of 2.0±0.8 (mean ± standard deviation) in children and 2.3±0.6 in adults. The image quality in patients with Formula stents was 2.3±1.2. The Cheatham Platinum stents induced 34–48% blooming, the Formula stents 44–55%. The image quality in patients with the less commonly used Atrium Advanta V12, IntraStent, AndraStent and Palmaz stents was scored 3 (good) to 4 (excellent) with less blooming. The electrocardiographic gating and tube voltage (kVp) did not affect image quality.

**Conclusions:**

There is a substantial variation in CT image quality and blooming artefacts for different stent types used to treat aortic coarctation.

## Key points

- Stent implant is the preferred treatment for aortic coarctation
- Contrast-enhanced CT is the modality of choice for follow-up and complications evaluation
- Different stent types demonstrated different image quality
- Electrocardiographic gating and tube voltage (kVp) did not affect image quality

## Background

Aortic stents are commonly used to treat aortic coarctation in both children and adults [1]. Contrast-enhanced computed tomography (CT) is often performed shortly after implantation to assess if complications have occurred during placement (e.g. dissection) and to evaluate the stent position. CT can also be used during follow-up to detect long-term complications such as in-stent stenosis, intimal hyperplasia, stent displacement and aneurysm formation [2]. According to European guidelines [3], imaging of the aorta should be performed after intervention to document post-implantation anatomy and detect possible complications, with the ideal imaging interval for follow-up depending on the exact baseline pathology. American guidelines [4] recommend follow-up with CT or magnetic resonance imaging (MRI) at intervals of five years or less after stent placement. Although both MRI and CT can be used [5], CT is often the modality of choice since in-stent assessment with MRI can be hampered by artefacts [6].

Several stent types are commercially available and used [7]. Stents were traditionally made of stainless steel, but nowadays different stent materials like cobalt-chromium and platinum-iridium alloys are used as well. It is known that the stent material has a large influence on image quality and stent lumen assessment with CT in coronary stents [8]. However, studies investigating the effect of different stent types on CT image quality for coarctation stents are lacking. Therefore, in this multicentre study the image quality as well as the presence and extent of blooming artefacts generated by different types of aortic stents on CT images were evaluated in patients treated for aortic coarctation.

## Methods

This study was performed at three tertiary centres in two different countries. At each centre, the study was approved by the local institutional review boards (protocol numbers 16/243/C, MEC-2016-281 and 290REG2016, respectively). A waiver for the requirement for informed consent was granted at all institutions because the study only involved analysis of previously acquired data.

### Patients

Patients with aortic coarctation who underwent a catheterisation procedure between 2003 and 2016 were included using the local catheterisation registries. Patients in whom an aortic stent was placed as well as patients who underwent re-dilatation of a previously implanted aortic stent were selected. Subsequently, a picture archiving and communication system (PACS) was searched to select patients who underwent contrast-enhanced CT angiography after implantation. In case multiple follow-up scans were available for a patient, only the first scan after implantation was analysed. Exclusion criteria were an unknown stent type and multiple overlapping stents. For each patient, sex, date of stent implantation, weight and height at implantation and stent type were recorded. Coded data were used for analysis.

**Fig. 1:**
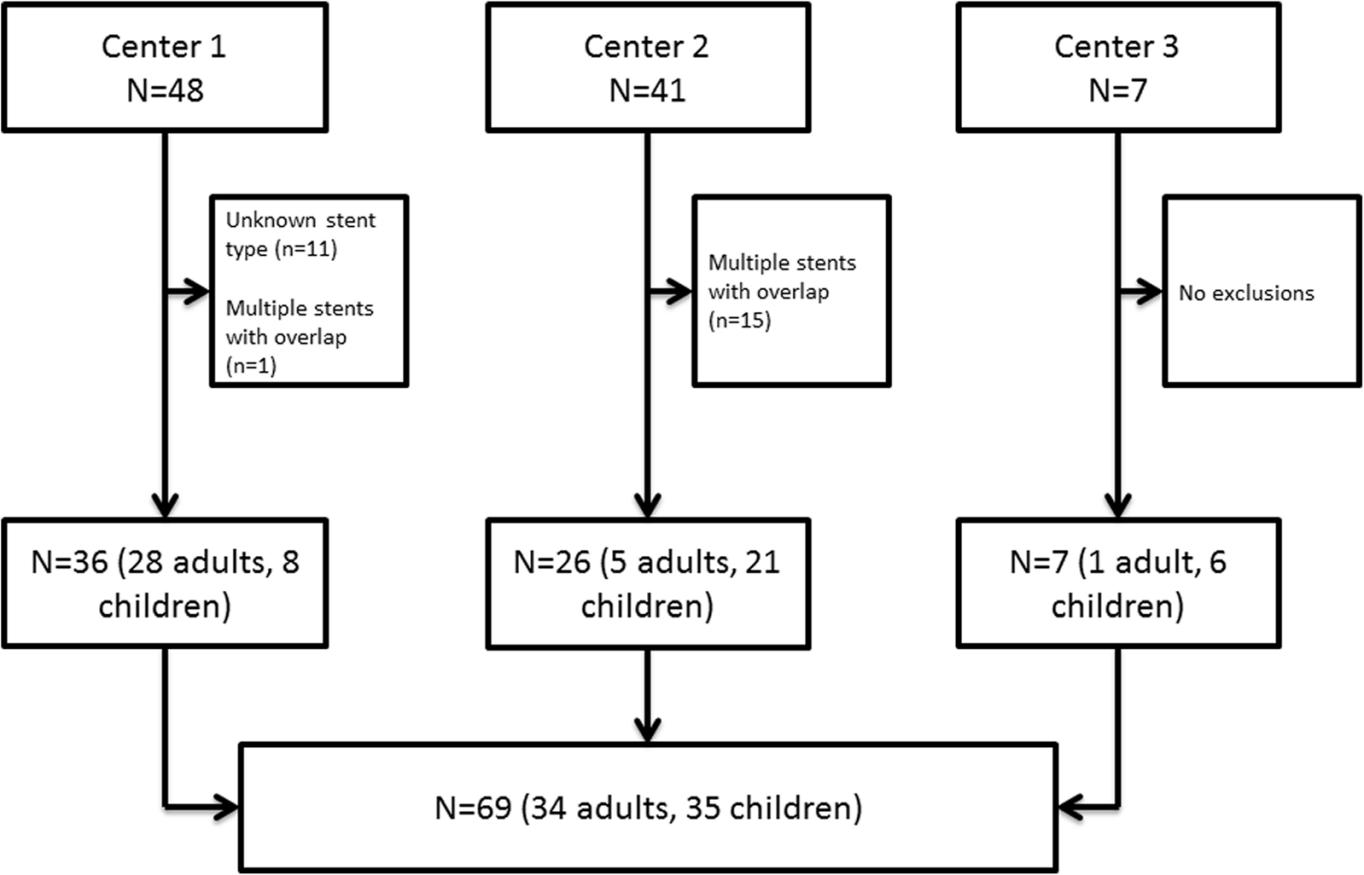
Flowchart of study inclusion

A study flowchart is provided in Fig. 1. In total 96 patients were eligible in three different centres. Eleven patients were excluded because the stent type was unknown, and 16 patients were excluded because multiple overlapping stents were present. Therefore, 69 patients were included of whom 35 were 18 years or younger at the time of stent implantation (paediatric patients).

### CT protocols

For all acquisitions, the following parameters were recorded: date; whether or not electrocardiographic gating or triggering was used; heart rate; tube current in milliamperes (mA); tube peak voltage (kVp); volumetric CT dose index; dose length product; CT system; and reconstruction slice thickness. The CT protocols adopted at each centre are summarised in Table 1. Characteristics of the CT acquisitions in the paediatric and adult populations are displayed in Table 2.

**Table 1:** CT protocols per centre

	Centre 1 (*n* = 36)	Centre 2 (*n* = 26)	Centre 3 (*n* = 7)
Type of scanner (number of slices)			
Siemens Somatom Force (2 × 192)	6 (17%)	–	–
Siemens Somatom Drive (2 × 128)	2 (6%)	–	–
Siemens Somatom Definition Flash (2 × 128)	16 (44%)	–	–
Siemens Somatom Definition AS+ (2 × 64)	8 (22%)	–	–
Siemens Sensation 64 (64)	2 (6%)	–	6 (86%)
Siemens Sensation 16 (16)	2 (6%)	–	–
Philips Brilliance iCT (2 × 128)	–	25 (96%)	1 (14%)
Philips Brilliance 16 (16)	–	1 (4%)	–
Total	36 (100%)	26 (100%)	7 (100%)
ECG gating	18 (50%)	21 (%)	–
Heart rate (bpm)	79±29	76±21	–
Tube current (mA)	198±126	176±66	80±33
Tube peak voltage (kVp)			
70	3 (8%)	–	–
80	3 (8%)	8 (%)	1 (14%)
90	3 (8%)	1 (%)	–
100	9 (25%)	11 (%)	5 (71%)
120	17 (47%)	6 (%)	1(14%)
140	1 (3%)	–	–
Total	36 (100%)	26 (100%)	7 (100%)
Volume CT dose index (mGy)	5.5±5.2	8±5	3.8±3
Dose length product (mGy × cm)	190±178	255±277	132±96
Slice thickness			
0.8	8 (22%)	–	5 (71%)
0.9	–	12 (50%)	1 (14%)
1	26 (72%)	2 (77%)	1 (14%)
1.5	–	12	–
2	2 (6%)	–	–
Total	36 (100%)	26 (100%)	7 (100%)
Kernel			
B20f	7	–	–
B26f	3	–	–
B31f	4	–	–
B40f	–	–	5
B46f	1	–	–
Bv40d	6	–	–
I26f	16	–	–
A	–	8	
B	–	6	1
C	–	1	–
CB	–	2	–
XCA	–	4	–
XCC	–	5	–
Total	36 (100%)	26 (100%)	7 (100%)

*bpm* beats per minute, *CT* computed tomography, *ECG* electrocardiogram

Data are frequencies and percentages or means ± standard deviations

**Table 2:** CT characteristics in paediatric and adult cases

	Paediatric cases (*n* = 35)	Adult cases (*n* = 34)
Time between implantation and CT		
< 1 month	12 (32%)	28 (82%)
1 month – 1 year	15 (41%)	3 (9%)
> 1 year	10 (27%)	3 (9%)
ECG gating	19 (54%)	20 (59%)
Heart rate (bpm)	82±28	73±21
Tube current (mA)	135±75	219±114
Tube peak voltage (kVp)		
70	3 (9%)	0 (0%)
80	10 (29%)	2 (6%)
90	2 (6%)	2 (6%)
100	15 (43%)	10 (29%)
120	5 (14%)	19 (56%)
140	0 (0%)	1 (3%)
Volume CT dose index (mGy)	4.9±3.8	7.7±6.0
Dose length product (mGy × cm)	128±128	292±266
Slice thickness ≤ 1 mm	28 (80%)	27 (79%)
Type of scanner		
16-slice	1 (3%)	2 (6%)
64-slice	5 (14%)	3 (9%)
> 64 slice	29 (83%)	29 (85%)

*bpm* beats per minute, *CT* computed tomography, *ECG* electrocardiogram

Data are frequencies and percentages or means ± standard deviations

### Image quality assessment

The overall subjective image quality of both the aortic lumen and the aortic wall at stent level was scored by one radiologist (SB) with five years of CT experience. The aortic wall was scored at three levels (proximal, central and distal third of the stent). Overall subjective image quality of the aortic lumen at the stent level was scored as (1) unacceptable, non-diagnostic image quality; (2) poor, limited diagnostic value; (3) good, diagnostic image quality; or (4) excellent, optimal diagnostic image quality. Subjective image quality of the aortic wall at stent level was assessed as (1) unacceptable, non-diagnostic image quality of the aortic wall, aortic wall not assessable due to severe artefacts or excessive noise; (2) poor, image quality of the aortic wall with limited diagnostic value, aortic wall is assessable but partially obscured due to moderate artefacts or noise; (3) good, diagnostic image quality of the aortic wall, possible to diagnose/exclude aortic wall abnormalities with minor artefacts or noise; or (4) excellent, excellent image quality of the aortic wall, possible to diagnose/exclude aortic wall abnormalities without artefacts or noise. Furthermore, it was evaluated if the stent was adjacent to the aortic wall. If there was a distance of ≥ 5 mm between the proximal and/or distal part of the stent and the aortic wall, the stent was judged as not adjacent.

A random sample of one third of the cases (*n* = 23) was scored twice by the same observer with an interval of at least two months in between to assess intra-observer variability. The observer was blinded to the previous scores. The same sample was scored by a second observer (RB), a radiologist with ten years of experience in cardiovascular imaging, to assess inter-observer variability. The second observer was blinded to the results of the first observer.

Objective image quality was evaluated by one observer with three years of CT experience (AH) using a bone setting (window width 1600, window level 300). A region of interest (ROI) was placed in the aorta at the level of the pulmonary trunk using thin slice reconstructions with a dedicated angiographic kernel. The ROI was drawn as large as possible without including the vessel wall. In the same slice an ROI was placed in the muscle tissue. Noise was defined as the standard deviation (SD) in Hounsfield units (HU) of the ROI. The signal-to-noise ratio was defined as the ratio between the mean HU and the SD of the same ROI. The contrast-to-noise ratio was calculated using the following equation [8–10]:

$$ CNR=\frac{{\mathrm{HU}}_{\mathrm{contrast}\ \mathrm{aorta}}-{\mathrm{HU}}_{\mathrm{muscle}}}{\sqrt{\ \frac{1}{\ 2}\ \left({{\mathrm{SD}}_{\mathrm{constrast}\ \mathrm{aorta}}}^2+{{\mathrm{SD}}_{\mathrm{muscle}}}^2\right)}} $$

The area-derived stent diameter was measured at three levels at the proximal, central and distal thirds of the stent using a bone setting (window width 1600, window level 300). Multiplanar reformations were used to obtain a cross-sectional plane of the stent where the measurements were taken. Both the inner and the outer stent diameters were derived. Stent struts can appear thicker than they are in reality due to metal-related artefacts such as blooming, scatter and partial volume averaging, which may lead to the spurious appearance of obstruction of the stent lumen on CT images. For simplification we will refer to this type of artefact as blooming, which was calculated by the following formula [5]:

$$ \mathrm{Blooming}=\frac{\mathrm{Measured}\ \mathrm{outer}\ \mathrm{stent}\ \mathrm{diameter}-\mathrm{Measured}\ \mathrm{inner}\ \mathrm{stent}\ \mathrm{diameter}}{\mathrm{Measured}\ \mathrm{outer}\ \mathrm{stent}\ \mathrm{diameter}}\times 100\% $$

Furthermore, the influence of stent diameter on image quality was assessed. For this purpose, the mean of the outer and inner diameters was used, averaged over three measurements (proximal, central, distal). We assessed if the subjective image quality and the aortic wall image quality were significantly different between electrocardiogram (ECG)-gated and non-ECG-gated acquisitions and if the image quality differed for different tube voltage or current (mA) levels.

Objective image analysis was performed using PACS viewers. Subjective image analysis and diameter measurements were performed locally using the PACS at one institution and centrally with the same multimodality workstation with multiplanar reconstructions for the other two centres.

### Assessment of complications

Each scan was assessed regarding the presence of any anomalies/complications in regard to the stent structure and position, the aortic wall and aortic branches. The official reports were also retrospectively evaluated. Thereafter, patients’ files were retrieved to assess if the changes were already mentioned in the description of the procedure and if the patient underwent further examination that confirmed the CT findings. Furthermore, medical records were checked to verify if any change in patient management directly related to the CT findings had been undertaken.

### Statistical analysis

SPSS version 21.0 for Windows (IBM, Armonk, NY, USA) and RStudio version 1.0.153 (RStudio, Inc., Boston, MA, USA) were used for statistical analysis. Values are displayed as mean ± SD unless stated otherwise. Differences in scores between stent types were assessed with the Mann-Whitney *U* test. The influence of ECG gating, kilovoltage and milliamperes on image quality was tested using the χ^2^ test, Fisher’s exact test and the Kruskal-Wallis test. The correlation between blooming artefacts and kilovoltage and milliamperes was investigated with the Spearman correlation coefficient. A *p* value below 0.05 was considered statistically significant. To assess the intra-observer agreement and reliability, the percentage of agreement and the quadratic weighted κ were calculated. For the remaining data, descriptive analysis was used.

## Results

### Patient characteristics

**Fig. 2:**
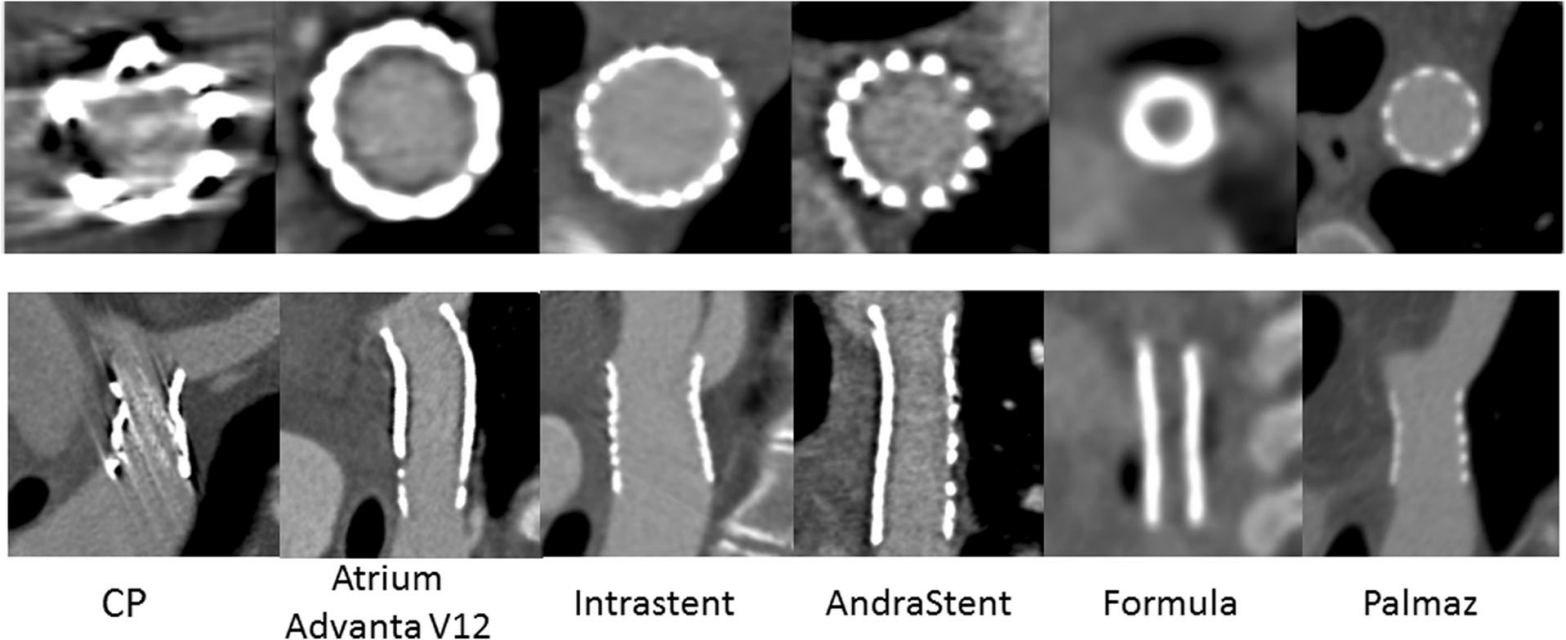
Overview of the different stent types. All images were displayed using a bone setting (window width 1600, window level 300), which was also used for image quality assessment. *CP*, Cheatham Platinum

Baseline patient characteristics are provided in Table 3. All adults had the Cheatham Platinum stent (NuMed Inc., Hopkinton, NY, USA) implanted, while different stent types were used in the paediatric patients, namely Cheatham Platinum (*n* = 18), Atrium Advanta V12 (*n* = 5, Atrium, Maquet Holding B.V. & Co. KG, Rastatt, Germany), IntraStent (*n* = 4, EV3 Inc., Plymouth, MN, USA), Formula (*n* = 3, Cook Medical, Bloomington, IN, USA), AndraStent (*n* = 4, Andramed GmbH, Reutlingen, Germany) and Palmaz (*n* = 3, Johnson & Johnson, New Brunswick, NJ, USA). The characteristics of the different stents are provided in Table 4. An example of CT images of the different stents is provided in Fig. 2. Two paediatric patients had two stents which were not overlapping. The mean stent diameter was 15±4 mm.

**Table 3:** Patient characteristics

	Paediatric cases (*n* = 35, 37 stents)	Adult cases (*n* = 34)
Males	20 (57%)	17 (50%)
Age (years)	10±5	42±16
Length (cm)	136±37	173±11
Weight (kg)	37±20	84±20
Type of stent		
Cheatham Platinum	18 (49%)	34 (100%)
Atrium Advanta V12	5 (14%)	0 (0%)
IntraStent	4 (11%)	0 (0%)
AndraStent	4 (11%)	0 (0%)
Formula	3 (8%)	0 (0%)
Palmaz	3 (8%)	0 (0%)
Multiple stents		
2 stents	2 (6%)	0 (0%)

Data are frequencies and percentages or means ± standard deviations

**Table 4:** Stent characteristics

Stent type	Manufacturer	Material	Design
Cheatham Platinum	NuMed Inc.	Platinum-iridium and joints over brazed with gold	Closed-cell
Atrium Advanta V12	Atrium, Maquet Holding B.V. & Co KG	316 L stainless steel	Open-cell
IntraStent	EV3 Inc.	Stainless steel	Open-cell
AndraStent	Andramed GmbH	Cobalt-chromium	Hybrid (open- and closed-cell)
Formula	Cook Medical	316 L stainless steel	Open-cell
Palmaz	Johnson & Johnson	Stainless steel	Closed-cell

### CT characteristics

ECG gating was used in 54% of paediatric and 59% of adult patients. The heart rate was higher in paediatric patients: 82±28 beats per minute (bpm) compared to 73±21 bpm in adults. The tube current and tube peak voltage were lower in the paediatric population, resulting in a lower radiation dose. The majority of acquisitions (83% paediatrics, 85% adults) were performed on ≥ 64 slice scanners.

### Image quality

**Fig. 3:**
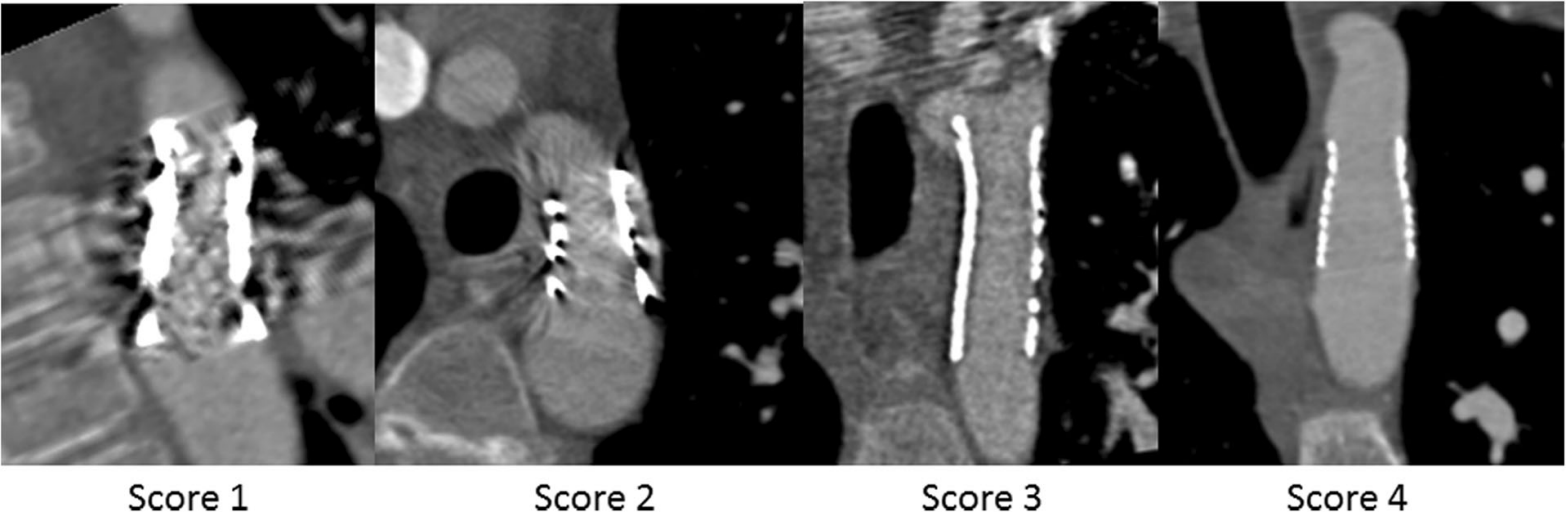
Examples of the scores from 1 to 4 for the aortic wall image quality. All images were displayed using a bone setting (window width 1600, window level 300), which was also used for image quality assessment

**Fig. 4:**
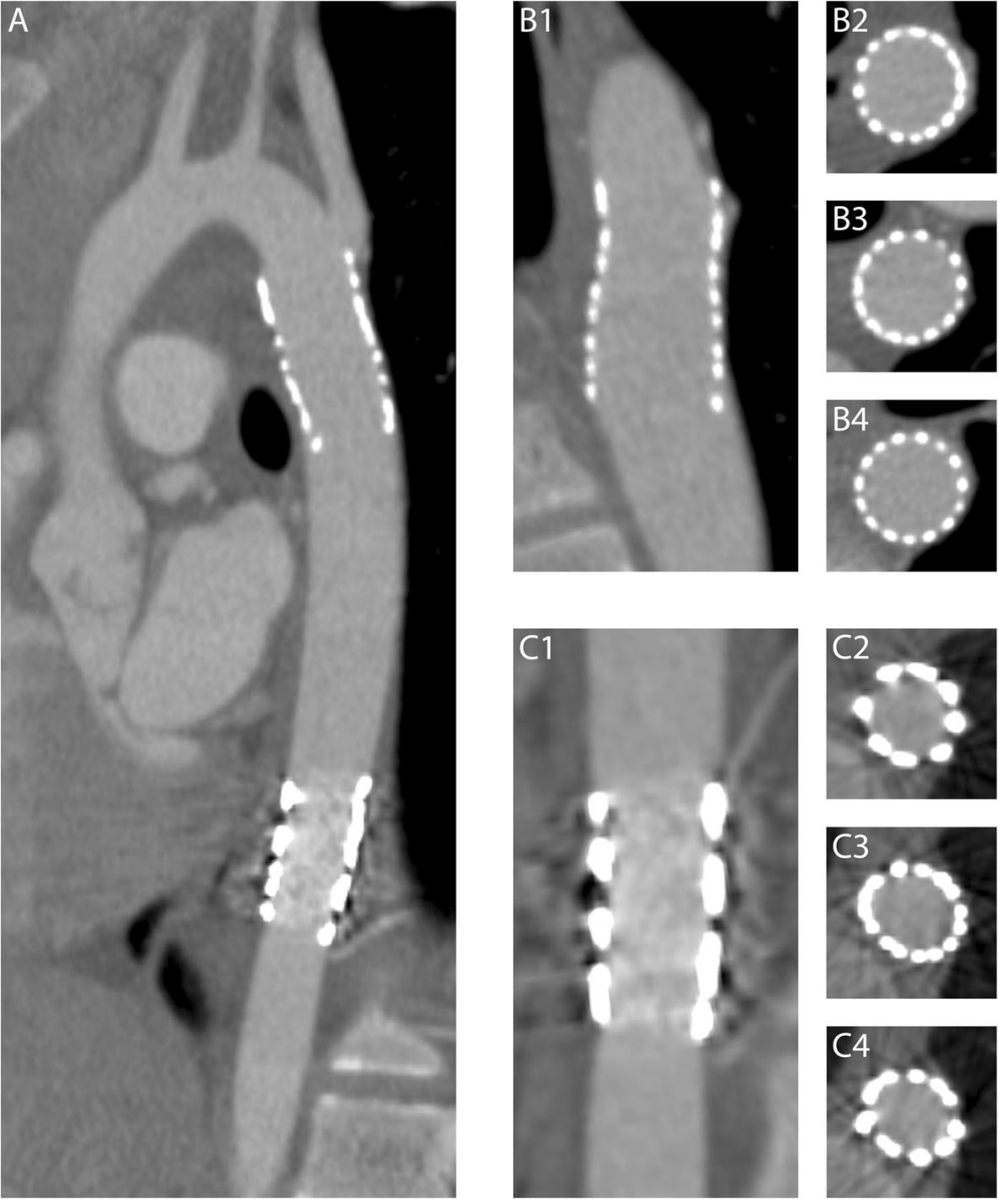
Differences in image quality between stents. Example illustrating the different image quality of two stents implanted in the same patient and, therefore, imaged with the same CT technical parameters (**a**). The more cranial stent (**b1**–**b4**) was an AndraStent that showed excellent quality of the lumen (**b1**) and of the aortic wall at stent level at the proximal (**b2**), mid (**b3**) and distal (**b4**) thirds of the stent. The more caudal stent was a Cheatham Platinum stent that showed lower image quality for both the lumen (**c1**) and the aortic wall at the three examined levels (**c2**, **c3**, **c4**)

Subjective and objective image quality results are provided in Table 5. Objective image quality was comparable between paediatrics and adults. The Cheatham Platinum and Formula stents had an overall subjective image quality of the lumen between 2 (poor) and 3 (good). Formula stents also had a relatively small stent diameter of 6±1 mm. Other stents scored good (score 3) to excellent (score 4), namely 3.4 (Atrium Advanta V12), 3.5 (IntraStent), 3.8 (AndraStent) and 4.0 (Palmaz). The results for the aortic wall image quality were similar, with poor scores for the Cheatham Platinum stent while the other stents scored good to excellent. When comparing Cheatham Platinum stents against each of the other stent types, the differences in subjective scores were statistically significant at all levels and for all stents (Advanta Atrium V12: lumen *p* = 0.003, proximal aortic wall *p* = 0.001, central aortic wall *p* < 0.001, distal aortic wall *p* = 0.050; Formula: proximal aortic wall *p* = 0.006, central aortic wall *p* = 0.002, distal aortic wall *p* = 0.019; IntraStent: lumen *p* = 0.001, proximal aortic wall *p* < 0.001, central aortic wall *p* < 0.001, distal aortic wall *p* < 0.001; Palmaz: lumen *p* < 0.001, proximal aortic wall *p* < 0.001, central aortic wall *p* < 0.001, distal aortic wall *p* = 0.001; AndraStent: lumen *p* < 0.001, proximal aortic wall *p* = 0.001, central aortic wall *p* < 0.001, distal aortic wall *p* = 0.003) with the exception of the image quality score of the lumen for the Formula stent (*p* = 0.564). An example of the different scores for the aortic wall image quality is provided in Fig. 3. In Fig. 4 a case example of a patient with both a Cheatham Platinum stent and an AndraStent illustrates the differences in image quality between the stents. Seventeen percent (12/71) of the stents were not completely adjacent to the aortic wall.

**Table 5:** Objective and subjective image quality per stent type

	Average stent diameter (mm)	Noise aorta (HU)	Noise muscle (HU)	Signal-to-noise ratio Aorta	Signal-to-noise ratio Muscle	Contrast to noise	Overall subjective image quality	Image quality aorta Proximal score	Image quality aorta Central score	Image quality aorta Distal score
Paediatrics										
Cheatham Platinum (*n* = 18)	14±3	30±13	24±10	13±7	3±2	12±6	2.0±0.8	2.3±0.7	2.0±0.7	2.4±0.8
Atrium Advanta V12 (*n* = 5)	11±3	28±16	23±8	13±2	3±1	11±3	3.4±0.9	3.6±0.5	3.6±0.5	3.6±0.5
IntraStent (*n* = 4)	12±4	44±21	26±9	12±7	3±1	13±6	3.5±0.6	4.0±0.0	4.0±0.0	4.0±0.0
AndraStent (*n* = 4)	17±3	32±17	32±23	15±6	3±1	14±7	3.8±0.5	3.8±0.5	3.8±0.5	3.8±0.5
Formula (*n* = 3)	6±1	30±17	15±5	10±2	6±1	9±1	2.3±1.2	3.7±0.6	3.7±0.6	3.7±0.6
Palmaz (*n* = 3)	15±1	29±16	28±13	17±11	3±1	14±8	4.0±0.0	4.0±0.0	4.0±0.0	4.0±0.0
Adults										
Cheatham Platinum (*n* = 34)	17±3	27±11	24±8	14±7	3±1	12±6	2.3±0.6	2.3±0.8	1.9±0.8	2.6±0.7

Data are means ± standard deviations. *HU* Hounsfield unit

The subjective image quality and the aortic wall image quality were not significantly different between ECG-gated and non-ECG-gated acquisitions. Also the tube voltage and current did not significantly alter the subjective image quality of the overall lumen (*p* = 0.703 and *p* = 0.425, respectively) or of the aortic wall at the proximal (*p* = 0.412 and *p* = 0.263, respectively) and distal thirds (*p* = 0.568 and *p* = 0.225, respectively). A weak influence on the image quality of the aortic wall at the level of the central third of the stent was found for the tube peak voltage (*p* = 0.038) but not for the tube current (*p* = 0.227).

The percentages of blooming per stent type are provided in Table 6. Blooming was highest for the Formula stent (55% at the centre of the stent). Also the Cheatham Platinum stents were associated with considerable blooming, namely 48% in paediatrics and 42% in adults. In the remaining stents, there was 22–37% blooming. In most stent types, blooming was higher at the centre of the stent compared to the outlets. A weak negative correlation was found between milliamperes and blooming artefacts at all levels of the stents (proximal, ρ = −0.345, *p* = 0.003; central, ρ = −0.299, *p* = 0.012; distal, ρ = −0.258, *p* = 0.031). No influence of tube peak voltage on blooming artefacts was found (proximal, *p* = 0.085; central, *p* = 0.285; distal, *p* = 0.122).

**Table 6:** Amount of blooming per stent type

	Percentage blooming proximal (%)	Percentage blooming central (%)	Percentage blooming distal (%)
Paediatrics			
Cheatham Platinum (*n* = 18)	42±7	48±10	35±15
Atrium Advanta V12 (*n* = 5)	34±13	37±11	32±7
IntraStent (*n* = 4)	29±7	36±12	31±11
AndraStent (*n* = 4)	24±1	23±5	22±6
Formula (*n* = 3)	47±5	55±5	44±13
Palmaz (*n* = 3)	24±4	22±5	30±19
Adults			
Cheatham Platinum (*n* = 34)	35±4	41±10	34±5

Data are means ± standard deviations

The influence of the stent diameter on the image quality was only assessed for the Cheatham Platinum stents, since only a limited number of stents were included for the other stent types. The results are provided in Table 7. A bigger stent diameter was associated with a higher subjective image quality and image quality of the aortic wall as well as less blooming.

**Table 7:** Influence of stent diameter on the subjective image quality, image quality of the aorta and the amount of blooming for the Cheatham Platinum stent. Stents implanted in adults (*n* = 34) and paediatrics (*n* = 18) were combined

	Subjective image quality	Image quality aorta Proximal score	Image quality aorta Central score	Image quality aorta Distal score	Percentage blooming proximal	Percentage blooming central	Percentage blooming distal
< 13 mm (*n* = 9)	1.8±0.7	1.7±0.4	1.7±0.5	1.9±0.3	45.8±5.8	51.1±10.0	40.5±4.3
13–15 mm (*n* = 11)	1.9±0.7	2.2±0.8	1.6±0.7	2.5±0.7	40.0±4.6	50.7±12.6	39.3±6.3
15–17 mm (*n* = 15)	2.1±0.5	2.1±0.6	1.9±0.7	2.6±0.7	36.7±3.7	41.3±5.0	32.2±1.5
17–20 mm (*n* = 10)	2.6±0.5	2.6±0.5	2.3±0.7	2.6±0.8	34.2±2.8	38.4±5.0	32.7±2.7
> 20 mm (*n* = 7)	2.7±0.5	3.0±1.0	2.4±1.1	3.1±0.7	29.7±2.0	33.9±4.2	27.5±2.3

Data are means ± standard deviation

### Intra- and inter-observer reliability and agreement

Intra-observer agreement for subjective image quality was 96%. Agreement for the image quality of the aortic wall at stent level was 96%, 87% and 79% for the proximal, central and distal parts of the stent, respectively. The intra-observer reliability was excellent with a weighted κ of 0.955 for the overall subjective image quality and 0.973, 0.928 and 0.848 for the image quality of the aortic wall for the proximal, central, and distal parts of the stent, respectively.

Inter-observer agreement for the subjective image quality of the lumen was 67%. Agreement for the image quality of the aortic wall at the proximal, central and distal parts of the stent was 63%, 63% and 42%, respectively. The inter-observer reliability was good with a weighted κ of 0.695 for the overall subjective image quality and 0.755 and 0.763 for the image quality of the aortic wall for the proximal and central parts of the stent, respectively. The inter-observer reliability for the image quality of the aortic wall at the level of the distal part of the stent was moderate with a weighted κ of 0.529. In no cases was the difference between the scores of the two observers more than one point.

### Detection of anomalies/complications

In total, 1 active bleeding, 1 pseudoaneurysm, 11 branches arising from the stent, 1 occluded branch, 2 cases of suspected intimal hyperplasia, 8 cases with focal stricture of the stent and 2 cases with both a stricture and a branch emerging from the stent were found.

In two cases the presence of a portion of the stent with a smaller diameter noticed on the CT scan had not been previously mentioned in the report of the stent implantation procedure. In one of these cases, as a consequence of the CT finding, the patient underwent an angiographic procedure that confirmed the restenosis, as well as its haemodynamic significance, and allowed subsequent re-dilatation. The procedural reports of the cases in which the active bleeding and the pseudoaneurysm were found on the follow-up CT scans indicated a regular and uneventful intervention. Following the CT scan, the first patient with a massive haemorrhage was operated upon but died the same day. For the second patient a conservative management was undertaken after the CT scan, and a later exam showed complete reabsorption of the pseudoaneurysm. The two cases with suspicion of intimal hyperplasia were not further investigated. In the other cases, the CT findings did not result in a modification of the patient management.

## Discussion

Coarctation of the aorta represents 5–10% of the cases of congenital heart disease [11]. In former times the only treatment option was surgery; however, since the 1980s transcatheter treatment with balloon angioplasty and, since the 1990s, stent implantation have evolved [11]. Data from the American College of Cardiology’s National Cardiovascular Data Registry of 671 transcatheter procedures in patients with aortic coarctation show that balloon angioplasty is the most common treatment (50.5%), followed by stent treatment (37.9%) or a combination of both (11.6%) [12]. Until March 2016, there were no US Food and Drug Administration (FDA)-approved stents for use in the aorta; therefore, stents were used off-label for this indication [13]. In 2016 the Cheatham Platinum stent obtained FDA approval, while the stent already had a CE mark for use on the European market [14]. A prospective multicentre trial involving 105 patients showed that the Cheatham Platinum stent is safe and effective [13]. Data on the safety and effectiveness of off-label stents are absent or limited to small retrospective studies [11, 15–17]. The choice to implant a specific type of stent relies on clinical and anatomical characteristics of the patients such as age, diameter of the aorta and of the stricture, previous operations/interventions, site of the lesion and anatomy of adjacent aorta and aortic branches [18].

There is consensus that follow-up imaging should be performed after stent implantation; however, the modality and the time interval are less clear [3, 4]. Early on, follow-up is necessary to rule out aortic rupture and delayed bleeding. Later on, other complications like delayed aneurysm formation, restenosis or fracture of the stent might occur. A consortium study with data from 34 centres and 302 patients showed that CT is the most often used imaging modality for follow-up [11]. Three months after implantation, 63% of patients had undergone a follow-up CT, while cardiac catheterisation (10%) and MRI (12%) were less commonly used. In 16% of patients no follow-up imaging was performed [11].

Although CT is commonly used for follow-up of stents implanted for aortic coarctation, data on the image quality of different stent types on CT are lacking. In this multicentre evaluation of the CT image quality in patients with aortic coarctation implanted with aortic stents, we showed that the most commonly used stent type (Cheatham Platinum) is associated with moderate but still diagnostic image quality on CT, while less commonly used stent types were associated with superior image quality. In addition, ECG gating and tube voltage did not demonstrate any effect on image quality.

The Cheatham Platinum stent consists of a platinum-iridium alloy and joints over brazed with gold. The other stent types are made of 316 L stainless steel (Atrium Advanta V12 and Formula), stainless steel (IntraStent and Palmaz) and a cobalt-chromium alloy (AndraStent). Our results regarding the Cheatham Platinum, i.e. the association with a worse but still diagnostic image quality, are in agreement with the in vitro study by Köhler et al. [19], who evaluated 22 different types of peripheral artery stents with CT angiography. A poorer image quality was reported for the platinum-iridium and tantalum stents due to blooming artefacts. They attributed this to the higher atomic number of platinum (78) and tantalum (73) compared to cobalt (27), steel (26) and chromium (24) [19]. Other factors which might have an effect on image quality besides the stent material are the stent diameter, strut design and acquisition parameters. In the current study we assessed the influence of stent diameter on the image quality for the Cheatham Platinum stent, and smaller stent diameters were associated with poorer image quality and a larger amount of blooming. This might also explain the results of the Formula stent, which was associated with moderate diagnostic image quality and substantial blooming. However, patients receiving the Formula stent were on average three years old; therefore, the smaller stent diameters used in those young children might explain the moderate image quality. The subjective image quality of Cheatham Platinum stents with a diameter below 15 mm was poor; however, the number of patients in this subgroup analysis was small. Although the image quality of Cheatham Platinum was poor for stents with a small diameter, CT might still be the preferred imaging modality for follow-up in small children because it is relatively non-invasive and adjacent organs can be assessed as well. Although the number of patients with different stent types was too small for analysis, the objective image quality of the scan did not seem to have an impact on the subjective image quality of the stent.

We found a weak negative correlation between tube current and percentage of blooming. Conversely, no effect of ECG gating and tube voltage on image quality was demonstrated, with the exception of a weak association between tube voltage and image quality of the aortic wall at the middle third of the stent. This might seem to contradict previous studies which reported a reduction in terms of metal artefacts by employing reconstructions with higher energy levels obtained from dual energy scans [20–22]. However, in our study the limited number of scans per kVp category and, especially, the additional differences in other scanning parameters are likely to have influenced the results. Moreover, our subjective scoring system was focussed on the diagnostic value of the image quality, which does not exactly correspond to the amount of artefacts [20]. Therefore, further studies with more standardised imaging protocols are necessary to establish if dual-energy scans and monochromatic reconstructions can improve the image quality of aortic stents.

This is the first large patient study that systematically compares the CT image quality of different stent types used for the treatment of aortic coarctation. It provides insights into differences between stent types and the associated amount of blooming. However, this study has limitations. First, the retrospective design implied that different CT systems and different protocols were used. As a consequence, it was not possible to study the influence of parameters like contrast medium, kernel and reconstruction technique due to the large heterogeneity of the collected data. However, this resembles the real-world situation in which differences between hospitals are common. Second, the number of patients receiving a stent other than the Cheatham Platinum was limited. Therefore, we could only assess the influence of the stent diameter for the Cheatham Platinum stents, since data for the other stent types were insufficient due to small numbers. Third, the true diameter to which the stent was inflated during angiography was often unknown. Therefore, it was not possible to investigate if the stent diameter measured on CT images differed from the true stent diameter. Finally, although the subjective score for image quality was designed to assess if the image quality was diagnostic, in this study we did not investigate the CT diagnostic accuracy in the absence of a reference test such as angiography performed at the same time. However, the occlusion of aortic branches and their origin from the stent as well as the presence of strictures of the stent frame were detected on CT images. Furthermore, the two most relevant complications occurring in this study population (active bleeding and pseudoaneurysm) were identified on CT examinations.

In conclusion, this study provides insights into the image quality and blooming artefacts of different stent types used to treat coarctation of the aorta on CT images. Both radiologists and clinicians should be aware of these differences when prescribing imaging exams, writing reports and interpreting the results. Radiologists should provide detailed information of the image quality of the CT scans in addition to the findings.

## Abbreviations

CT: Computed tomography
ECG: Electrocardiogram
HU: Hounsfield unit
MRI: Magnetic resonance imaging
PACS: Picture archiving and communication system
ROI: Region of interest
SD: Standard deviation

## References

[1] Eichhorn JG, Long FR, Hill SL (2006). Assessment of in-stent stenosis in small children with congenital heart disease using multi-detector computed tomography: a validation study. Catheter Cardiovasc Interv.

[2] Eichhorn JG, Long FR, Jourdan C (2008). Usefulness of multidetector CT imaging to assess vascular stents in children with congenital heart disease: an in vivo and in vitro study. Catheter Cardiovasc Interv.

[3] Baumgartner H, Bonhoeffer P, De Groot NM (2010). ESC Guidelines for the management of grown-up congenital heart disease (new version 2010). Eur Heart J.

[4] Warnes CA, Williams RG, Bashore TM (2008). ACC/AHA 2008 Guidelines for the Management of Adults with Congenital Heart Disease: a report of the American College of Cardiology/American Heart Association Task Force on Practice Guidelines (writing committee to develop guidelines on the management of adults with congenital heart disease). Circulation.

[5] den Harder AM, Sucha D, van Hamersvelt RW (2017). Imaging of pediatric great vessel stents: computed tomography or magnetic resonance imaging?. PLoS One.

[6] Jurcut RDA, Lorber A (2011). Coarctation of the aorta in adults: what is the best treatment? Case report and literature review. J Med Life.

[7] Maintz DSH, Raupach R (2003). Balloon expandable stents for coarctation of the aorta: review of current status and technical considerations. Eur Radiol.

[8] Maintz D, Seifarth H, Raupach R (2006). 64-slice multidetector coronary CT angiography: in vitro evaluation of 68 different stents. Eur Radiol.

[9] den Harder AM, Willemink MJ, Bleys RL (2014). Dose reduction for coronary calcium scoring with hybrid and model-based iterative reconstruction: an ex vivo study. Int J Cardiovasc Imaging.

[10] Szucs-Farkas Z, Strautz T, Patak MA, Kurmann L, Vock P, Schindera ST (2009). Is body weight the most appropriate criterion to select patients eligible for low-dose pulmonary CT angiography? Analysis of objective and subjective image quality at 80 kVp in 100 patients. Eur Radiol.

[11] Holzer R, Qureshi S, Ghasemi A (2010). Stenting of aortic coarctation: acute, intermediate, and long-term results of a prospective multi-institutional registry—Congenital Cardiovascular Interventional Study Consortium (CCISC). Catheter Cardiovasc Interv.

[12] Moore JW, Vincent RN, Beekman RH (2014). Procedural results and safety of common interventional procedures in congenital heart disease: initial report from the National Cardiovascular Data Registry. J Am Coll Cardiol.

[13] Meadows J, Minahan M, McElhinney DB, McEnaney K, Ringel R, Coast I (2015). Intermediate outcomes in the prospective, ulticenter Coarctation of the Aorta Stent Trial (COAST). Circulation.

[14] Zuckerman B (2016). FDA approval Cheatham Platinum stent.

[15] Venczelova Z, Tittel P, Masura J (2013). First experience with AndraStent XL implantation in children and adolescents with congenital heart diseases. Catheter Cardiovasc Interv.

[16] Bondanza S, Calevo MG, Marasini M (2016). Early and long-term results of stent implantation for aortic coarctation in pediatric patients compared to adolescents: a single center experience. Cardiol Res Pract.

[17] Hak Lee Ang TCWL (2014) Coarctation of the aorta: nonsurgical treatment using stent implantation. Singapore Med J 55:302–304 10.11622/smedj.2014080 PMC4294052 25017404

[18] Boccalini S, den Harder AM, Witsenburg M (2017). Complications after stent placement for aortic coarctation: a pictorial essay of computed tomographic angiography. J Thorac Imaging.

[19] Köhler M, Burg MC, Bunck AC, Heindel W, Seifarth H, Maintz D (2011). Dual-source CT angiography of peripheral arterial stents: in vitro evaluation of 22 different stent types. Radiol Res Pract.

[20] Meinel FG, Bischoff B, Zhang Q, Bamberg F, Reiser MF, Johnson TR (2012) Metal artifact reduction by dual-energy computed tomography using energetic extrapolation: a systematically optimized protocol. Invest Radiol 47:406–414 10.1097/RLI.0b013e31824c86a3 22659595

[21] Secchi F, De Cecco CN, Spearman JV (2015). Monoenergetic extrapolation of cardiac dual energy CT for artifact reduction. Acta Radiol.

[22] Bamberg F, Dierks A, Nikolaou K, Reiser MF, Becker CR, Johnson TR (2011). Metal artifact reduction by dual energy computed tomography using monoenergetic extrapolation. Eur Radiol.

